# Use of LVAD HeartMate 3 and Impella RP in the management of advanced heart failure in an elderly patient: a case report

**DOI:** 10.1007/s12055-025-02071-x

**Published:** 2025-11-18

**Authors:** Maja Butrym, Kacper Jaros, Andrzej Juraszek, Tomasz Hrapkowicz

**Affiliations:** 1 Student Research Group at the Department of Cardiac Surgery, Transplantology, Vascular and Endovascular Surgery, Faculty of Medical Sciences in Zabrze, Medical University of Silesia, Katowice, Poland; 2 Department of Cardiac Surgery, Transplantology, Vascular and Endovascular Surgery, Faculty of Medical Sciences in Zabrze, Medical University of Silesia, Katowice, Poland

**Keywords:** Biventricular heart failure, HeartMate 3, Impella RP, Cardiac tamponade

## Abstract

Heart failure (HF) is a leading cause of morbidity and mortality. Mechanical circulatory support (MCS), such as the HeartMate 3 left ventricular assist device (LVAD), is a therapeutic option for patients who are ineligible for heart transplantation. Right heart failure (RHF), depending on the clinical criteria applied for its diagnosis, occurs in 10–40% of patients following LVAD implantation and significantly worsens prognosis. This report presents a 69-year-old male with chronic ischemic HF, prior cardiac resynchronization therapy with defibrillator (CRT-D) implantation, and coronary intervention with drug-eluting stents. Due to progressive mitral regurgitation (MR) and elevated HF biomarkers, MitraClip implantation was performed with initial clinical improvement. Subsequently, left ventricular function deteriorated despite optimized pharmacotherapy, leading to HeartMate 3 LVAD implantation. The postoperative course was complicated by recurrent cardiac tamponade requiring surgical management. Progressive RHF necessitated temporary support with an Impella RP device. Further complications included recurrent infections, sepsis, respiratory failure, and acute kidney injury requiring renal replacement therapy. Despite comprehensive treatment, progressive multiorgan failure and severe cachexia developed. Given the patient’s status as a destination therapy, care was transitioned to home-based palliative management. This case illustrates the complexity of managing end-stage HF with LVAD support, highlighting the frequent complications such as RHF and infections, and the importance of recognizing when to prioritize comfort in advanced disease.

## Introduction

Heart failure (HF) is associated with high morbidity and mortality, affecting nearly 65 million individuals worldwide, according to the European Society of Cardiology (ESC). Although significant progress has been made in the management of coronary artery disease and acute myocardial infarction (MI) over the past two decades, MI continues to be the leading contributor to the development of HF. In the absence of contraindications, heart transplantation (HTx) remains the standard of care for advanced HF, significantly improving prognosis and quality of life. Mechanical circulatory support (MCS) systems offer a therapeutic alternative, particularly when HTx is delayed or contraindicated (e.g., age > 65 years). These devices serve as both bridge-to-transplant (BTT) and destination therapy (DT) [1, 2].

HeartMate 3 is one of the most modern and widely used left ventricular assist devices (LVADs) [3]. A significant complication following LVAD implantation is right heart failure (RHF), which may develop in 10–40% of patients and is associated with increased early postoperative mortality [4]. In cases of secondary right ventricular failure (RVF) unresponsive to optimal pharmacologic therapy, the implementation of a percutaneous right ventricular assist device (RVAD) such as the Impella RP may constitute a viable and effective therapeutic strategy [5]. This short-term mechanical circulatory support system has been shown to facilitate rapid hemodynamic stabilization, augment cardiac output, improve end-organ perfusion, and support right ventricular (RV) functional recovery—factors that may collectively contribute to improved clinical outcomes in critically ill patients [6].

The patient discussed herein, with chronic HF of ischemic etiology resulting from coronary artery disease (CAD) with a history of myocardial infarction and percutaneous intervention with two drug-eluting stents (DES) placement in obtuse marginal (OM) branch 5 years before initial admission. A cardiac resynchronization therapy with defibrillator (CRT-D) device was implanted 7 years earlier due to persistent atrial fibrillation (AF), first-degree atrioventricular (AV) block, and right bundle branch block (RBBB). He also suffered from chronic kidney disease with severely reduced glomerular filtration rate (GFR). The patient’s final hospitalization, lasting 185 days at the age of 69, culminated in the initiation of palliative care. The clinical course over the years is outlined below.

## Case presentation

Initially, the patient was clinically stable without signs of congestion. At the admission, elevated N-terminal pro-B-type natriuretic peptide (NT-proBNP) levels (2702 pg/ml), severe left ventricular (LV) dysfunction (left ventricular ejection fraction (LVEF) 16%), slightly reduced RV function (tricuspid annular plane systolic excursion (TAPSE) 19 mm), impaired renal function (GFR 41 ml/min/1.73 m^2^), and significant mitral regurgitation (MR) and tricuspid regurgitation (TR) were noted. Cardiopulmonary exercise testing (CPET) revealed a maximal oxygen consumption (VO_2max_) of 16.0 ml/kg/min. Pharmacological therapy was optimized, and the patient was placed under close observation with regard to heart transplant eligibility.

Over the following months, the patient’s condition deteriorated, with resting dyspnea and increased NT-proBNP (7135 pg/ml). Electrocardiogram (ECG) showed LVEF 18%, severe MR, and signs of pulmonary hypertension (PH). Transesophageal echocardiography (TEE) confirmed proper valve anatomy, so the patient was qualified for elective MitraClip. Right heart catheterization revealed PH and RV dysfunction. Sildenafil therapy led to hemodynamic improvement, and he remained under close follow-up at the HF Day Clinic.

In subsequent years, an improvement in LVEF to 26–28% and resolution of PH were noted. MitraClip implantation with two clips (XTW, NTW) was performed without complications, resulting in significant MR reduction.

In later admissions, cardiac function worsened. LVEF declined to 15%, with moderate MR and TR. PH was not present. The patient was placed on the active heart transplant waiting list and remained stable under optimized pharmacotherapy.

In the following months, his condition progressively deteriorated. The patient developed reduced exercise tolerance, dyspnea, weight gain, and signs of ascites. LVEF progressively deteriorated (7–15%) with moderate-to-severe MR/TR and marked chamber dilatation. Although there were no overt clinical signs of advanced RV failure at that stage. ECG assessment indicated borderline RV function, as reflected by TAPSE of 19 mm and right ventricular fractional area change (RVFAC) of 27%. Based on these findings, the patient was qualified for LVAD implantation.

**Fig. 1:**
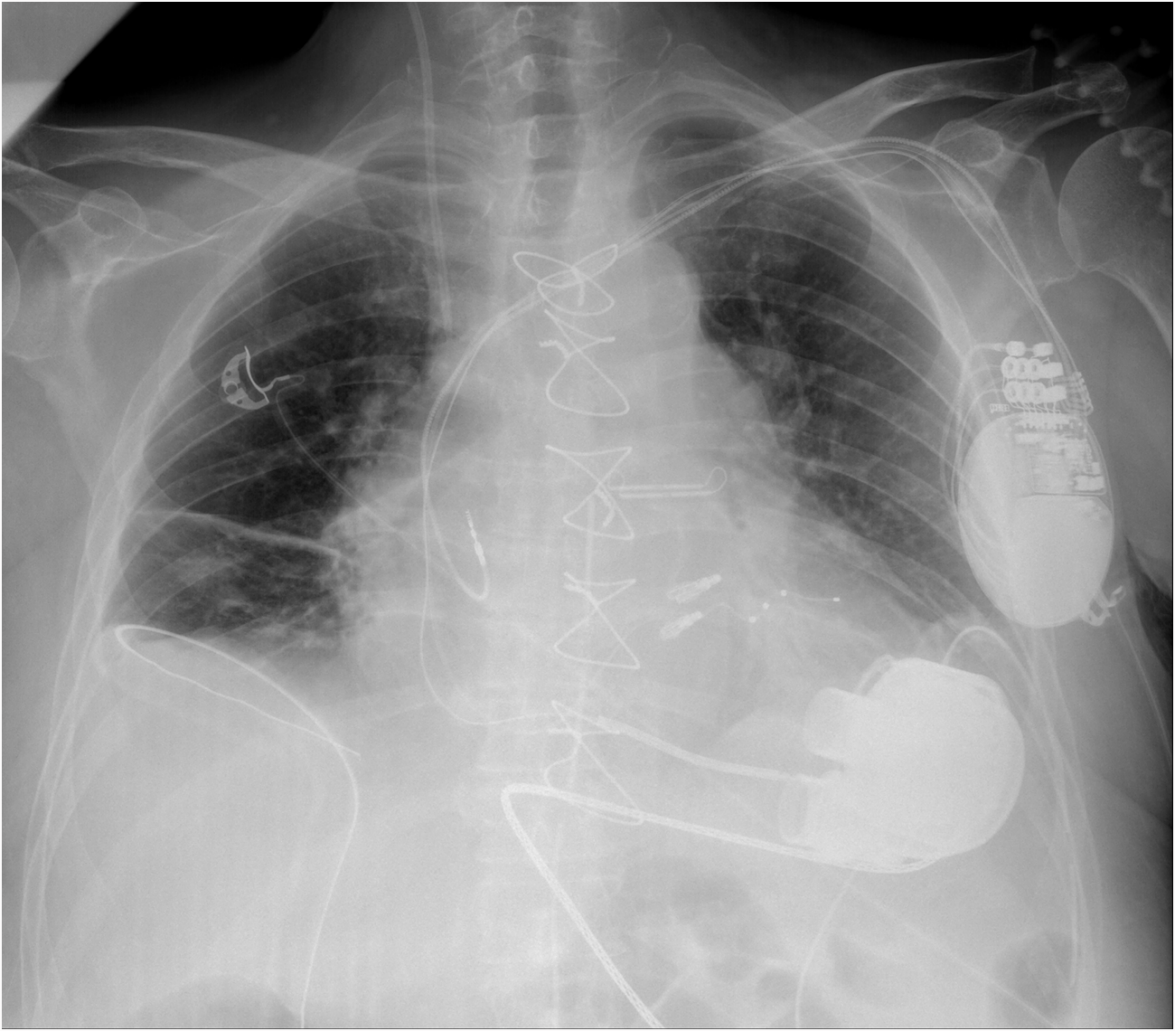
Chest X-ray showing the cardiac silhouette, CRT-D system leads and device, implanted two MitraClip clips, and the HeartMate 3 LVAD. CRT-D, cardiac resynchronization therapy with defibrillator; LVAD, left ventricular assist device

The HeartMate 3 LVAD was implanted along with closure of an atrial septal defect (ASD) and the left atrial appendage (Fig. 1). Postoperatively, the patient remained intubated, requiring pharmacological support with adrenaline, noradrenaline, and milrinone. In the following days, gradual clinical improvement allowed tapering of catecholamines, chest drain removal, and initiation of rehabilitation. During this period, progressive activated partial thromboplastin time (aPTT) elevation led to discontinuation of heparin infusion. Simultaneously, progressive pericardial effusion with hematoma formation up to 30 mm was observed, ultimately necessitating surgical management. On the same day, reaccumulation of pericardial fluid resulted in recurrent cardiac tamponade, requiring immediate surgical intervention. Intraoperatively, the anastomotic sites were dry after evacuation of fluid and thrombi. The postoperative course was further complicated by a central line-associated infection, reflected by elevated inflammatory markers (C-reactive protein (CRP) 150 mg/l, leukocytosis), which required targeted antibiotic therapy.

**Fig. 2:**
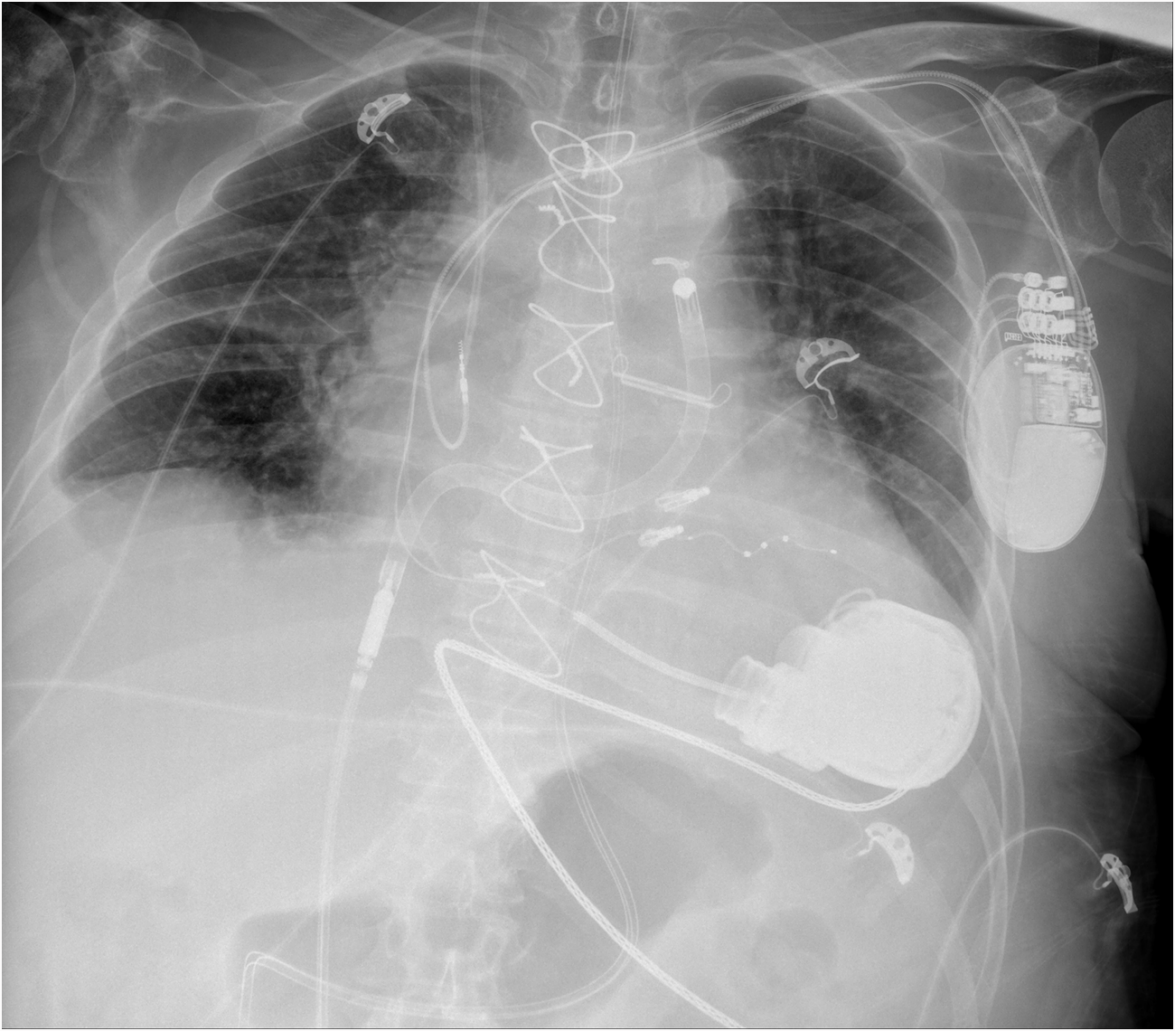
Bedside chest X-ray following implantation of the Impella RP system

These events, combined with the hemodynamic load of LVAD support, likely contributed to progressive RV deterioration. Despite improved LV function, severe RV failure with TAPSE of 10 mm and global hypokinesia necessitated temporary right-sided mechanical support with an Impella RP device (Fig. 2). The procedure was successful, resulting in hemodynamic stabilization, restoration of diuresis, improved organ perfusion, and general clinical improvement. As no RVAD-related complications were observed and device function remained stable, explantation was performed on day 18. However, persistent RV dysfunction required ongoing intensive pharmacological management. The clinical course was further complicated by recurrent infectious events, including a groin wound infection (post-cannulation), two episodes of urinary tract infections, and a septic episode, all of which were successfully managed with targeted antibiotic therapy. Regardless of optimal treatment, the patient’s condition continued to deteriorate, with worsening fluid overload, anuria, and multiorgan failure. Renal replacement therapy, intensive diuretic therapy, and repeated paracenteses were required. Acute respiratory failure necessitated re-intubation, mechanical ventilation, and subsequently tracheostomy. After stabilization, the patient could breathe independently, and a feeding gastrostomy was placed.

Given the patient’s destination therapy status, he was disqualified from long-term RVAD implantation. Management focused on pharmacological stabilization and ICD adjustments. Ultimately, despite transient clinical stability, progressive cachexia, lack of logical communication, and the palliative nature of care led to discharge for home-based palliative care. Active treatment was not escalated, allowing the patient to pass peacefully in the presence of his family. The timeline of the patient’s hospitalization and key interventions is summarized in Table 1.

**Table 1:** Timeline of hospitalizations and key clinical parameters

Date	Procedure	LVEF (%)	MR	TR	mPAP (mmHg)*
Feb 2017	CRT-D implantation	36	Mild	-	-
Jun 2019	DES implantation (PCI)	21	Mild	-	-
Oct 2019	Follow-up	16	Moderate	-	-
Apr 2020	Follow-up	17	Moderate	-	-
Oct 2020	Follow-up/pharmacologic treatment changes	18	Severe	Mild	31
Jan 2021	Follow-up	26	Severe	Moderate	10
Dec 2021	Follow-up	28	Severe	Moderate	-
May 2022	MitraClip implantation	21	Severe → mild	Moderate	-
Dec 2022	Follow-up	18	Moderate	Moderate	-
Jun 2023	Follow-up	15	Moderate	Moderate	18
Jan–Aug 2024	HTx qualification, LVAD + Impella RP implantation	7–15**	Moderate/severe**	Moderate/severe**	31***

*MR*, mitral regurgitation; *TR*, tricuspid regurgitation

*If right heart catheterization was performed

**At admission

***First right heart catheterization performed during the initial hospitalization

## Discussion

This patient’s clinical course illustrates the complex therapeutic challenges associated with managing advanced HF in elderly transplant-listed patients. Although eligible for HTx, his advanced age reduced his chances of receiving a donor organ promptly, lowering his priority on the waiting list. Progressive hemodynamic deterioration necessitated mechanical circulatory support, ultimately transitioning to destination therapy.

The implantation of the HeartMate 3 LVAD was complicated by two episodes of cardiac tamponade, both surgically managed on the same day. Anticoagulation after LVAD implantation increases the risk of postoperative bleeding and surgical reintervention, both linked to worse outcomes. One such potentially life-threatening consequence is cardiac tamponade, which may present either overtly or with subtle clinical signs and should always be considered in the differential diagnosis of hemodynamic instability in the early postoperative period. However, in this case report, prolonged aPTT persisted despite withdrawal of heparin therapy due to the cardiac tamponade suspicion, suggesting the possibility of an intrinsic coagulopathy or bleeding tendency. Pericardial effusion, frequently observed in patients receiving anticoagulation, further underscores the need for vigilant monitoring and prompt intervention [7, 8].

Later during hospitalization, despite improved LV hemodynamics, persistent RV dysfunction prompted implantation of short-term RV support using the Impella RP system. Although RHF is a known complication of LVAD therapy, reported incidence varies widely, ranging from 10 to 40% [4]. In the MOMENTUM 3 trial, the 5-year follow-up for the HeartMate 3 device showed an incidence of 29.9% [9]. Due to progressive RVF, around 10% of patients receiving LVAD will eventually require RVAD support, although anticipating this necessity before or during surgery remains a significant challenge [4].

Given the patient’s unstable clinical condition, prior tamponade episodes, and progressive circulatory failure, the less invasive percutaneous implantation of the Impella RP was chosen. Short-term use of the Impella RP can serve as a bridge to RV recovery in early postoperative LVAD patients. However, management of persistent late RVF remains a major therapeutic challenge [10, 11]. In this case, use of the Impella RP improved organ perfusion and contributed to amelioration of renal failure symptoms.

Despite transient hemodynamic stabilization and successful device explantation, persistent RVF and recurrent infections significantly worsened the patient’s overall prognosis. In view of the prolonged course of treatment, the advanced stage of HF, and the absence of realistic prospects for recovery, the decision was made to initiate palliative care. In our opinion, this approach was the most appropriate and ethically justified in the given clinical context.

## Conclusions

This case illustrates several clinically significant considerations in the management of advanced HF patients undergoing mechanical circulatory support. Despite meeting transplant criteria, patients over the age of 65 years face significantly reduced access to donor hearts, emphasizing the essential role of durable MCS as destination therapy in this demographic. The HeartMate 3 LVAD provided effective circulatory support; however, the clinical course was complicated by life-threatening events such as cardiac tamponade and infection, reflecting the complexity and risk profile of LVAD therapy. Post-LVAD RVF remains a major challenge, occurring in an estimated 10% of recipients. It often develops despite thorough preoperative assessment, necessitating preparedness for rapid escalation, including the use of RVADs. The Impella RP device served as a temporary support for RV recovery and systemic perfusion. Although its effects may be transient, it can offer a critical window for stabilization in the early postoperative period. When multi-organ failure progresses despite full mechanical support, transitioning to palliative care may represent the most ethical and patient-centered approach, particularly in the absence of transplant candidacy and reversibility of organ dysfunction.

## Data Availability

Not applicable.
